# Functionally disconnected: A look at how study design influences neurofeedback data and mechanisms in attention-deficit/hyperactivity disorder

**DOI:** 10.1371/journal.pone.0200931

**Published:** 2018-08-10

**Authors:** Justin Hudak, David Rosenbaum, Beatrix Barth, Andreas J. Fallgatter, Ann-Christine Ehlis

**Affiliations:** 1 LEAD Graduate School & Research Network, University of Tübingen, Tübingen, Germany; 2 Department of Psychiatry and Psychotherapy, University Hospital Tübingen, Tübingen, Germany; 3 Centre for Integrative Neuroscience, University of Tübingen, Tübingen, Germany; 4 Graduate School of Neural and Behavioral Sciences, University of Tübingen, Tübingen, Germany; University of Zurich, SWITZERLAND

## Abstract

Neurofeedback (NF) is a form of behavioral therapy used to treat e.g. attention-deficit/hyperactivity disorder (ADHD). Briefly, subjects are fed-back a putatively dysfunctional parameter of their brain activity in real time and must learn to control it in a suggested direction. NF protocols for ADHD have been used in practice for decades, though no clear standards on NF design have been implemented. Furthermore, studies often present only data from the general outcome of the NF treatment and do not look at how exactly the NF paradigm affects brain functionality, or what exactly the NF is training. The current study is two-fold: firstly, we look at how the functional connectivity (FC) patterns within key networks associated with ADHD differ between rests, feedback trials, success and failure in a complete functional near-infrared spectroscopy-based NF experiment on adults with ADHD. Secondly, due to methodological concerns discovered during the analysis of our data, we address important considerations in the design of NF that are often ignored in protocols being used widely in therapy and research today. In particular, we examine the common average reference and its impact on network activity as well as the importance of balancing the randomization in a design. Finally, we discuss how these methodological considerations may have influenced our FC results.

## Introduction

Broadly, neurofeedback (NF) is a form of behavioral therapy in which participants must learn to control a particular parameter of their brain activity by monitoring this parameter in real time via auditory, visual or combined feedback. NF therapy has become ubiquitous in modern times, developed for everything from enhancing cognitive activity in healthy populations to treating tinnitus [1,2]. However, NF is a contentious topic in current neuroscientific research [3]. The contention regarding NF protocols arises from the complexity of the human brain; human behavior is normally not based on any one parameter of activity, such as NF designs tend to target, but rather on a complex interplay of different brain structures and brain frequencies [4]. NF is now commonly used to treat attention-deficit/hyperactivity disorder (ADHD), a prevalent and disruptive disorder that affects roughly 2.5–5% of adults worldwide [5,6]. Symptoms include abnormal levels of impulsivity, hyperactivity, and inattention. NF with ADHD mirrors the greater NF community in that there are myriad protocols available to treat the same condition, each based on different theories about the neurobiology of the disorder.

Theta-beta frequency-band NF, slow cortical potential (SCP)-based NF, real-time functional magnetic resonance imaging NF (rt-fMRI), and functional near-infrared spectroscopy-based (fNIRS) NF have all been used in studies seeking to treat the behavioral alterations of both adult and juvenile ADHD [7–11]. Effect sizes for treatment are inconclusive, with earlier data suggesting medium to strong effects based on all prospective controlled [12] or only randomized trials [13]. However, a recent meta-analysis by Cortese et al. (2016) concluded that when NF designs are randomized, sham-controlled and double-blinded, the so-called gold standard for NF, they are not conclusively more effective than sham in treating ADHD. They further cite a lack of standardized protocols and the participants’ failure to learn the feedback as potential pitfalls concerning the design of the study. It is estimated, for example, that 15–30 percent of participants fail to learn control over the feedback parameter in every feedback study [14], whereby it is assumed that successful learning of control over the feedback parameter will result in the desired behavioral modification [15]. The standardization of each type of ADHD NF protocol, i.e. having the same number of trials, the same distribution of activation/deactivation trials, the same brain region or electrode targeted, would allow for better evaluation of small changes made between the protocols to advance the state of NF with ADHD. Finally, most NF studies in ADHD focus solely on clinical outcome measures, while only a few have additionally looked at how NF paradigms affect brain functionality [16,17] or what exactly is being trained during NF trials. The present study, therefore, focuses on brain activation changes during successful and failed feedback trials and the preceding resting periods, whereby the interplay between single brain regions is specifically considered.

ADHD is considered to be a disorder of network dysfunction on a large scale [18]. Affected networks seem to be as diverse as the symptoms belonging to the disorder itself. Castellanos and Proal [19] identify seven different cognitive networks associated with deficits in ADHD compared to healthy controls, which encompass nearly the entirety of the cortex. The prefrontal cortex, an area widely associated with executive functioning, is typically under-active or under-developed in childhood and adult ADHD [20]. Furthermore, this region is vital to a brain network called the frontoparietal control network (FPCN) where it assumes connections with frontal, striatal, motoric and parietal regions to restrain impulsive behavior and allow focus on cognitively strenuous tasks.

The interplay between the default mode network (DMN) and the FPCN is perhaps the most relevant to task-based behavior in ADHD. In healthy controls, the DMN [21], composed of medial prefrontal cortex, precuneus, and parietal areas, demonstrates strong functional connectivity (FC) during resting states. Furthermore, healthy controls exhibit strong FC in the FPCN during tasks requiring a great deal of cognitive control, and when healthy controls switch between cognitively demanding tasks and rest, there is a clear switch of responsible network. FC is typically anti-correlated in the FPCN and the DMN when cognitive tasks versus resting state are compared [18,21–23]. In individuals with ADHD however, this switch is less clear or non-existent, the FPCN failing to switch to the DMN during rest and vice-versa [18]. This failure to switch causes many problems, namely failure to rest during resting periods and failure to sustain attention [24] or increased errors during cognitive tasks.

A newly emerging NF protocol for ADHD utilizes functional near-infrared spectroscopy (fNIRS) to feedback oxygenated hemoglobin (O_2_Hb) activity from the prefrontal cortex, an area traditionally implicated in the disorder [8–10]. O_2_Hb activity reflects activation of the underlying brain region and is the chromophore most strongly correlated with the blood-oxygenation level-dependent (BOLD) response synonymous with functional magnetic resonance imaging (fMRI) studies [25]. fNIRS is an optical imaging method that takes advantage of the special properties of near-infrared light, and its interplay with the human skull and brain matter, to image cortical activation. fNIRS affords several advantages to more traditional imaging methods such as EEG and fMRI. In particular, fNIRS provides spatial resolution that is higher than that of EEG raw data and temporal resolution that is higher than that of fMRI, balancing it nicely between the two methods when considering NF experiments [26]. It allows access to cortical hemodynamics in a similar manner to fMRI, but is much cheaper, has a much easier subject preparation phase, and allows the subject to sit in a relatively naturalistic setting, such as a comfortable chair. The recent development of portable fNIRS devices makes the potential for ecological validity greater than ever. Furthermore, fNIRS is less susceptible to motion artifacts than EEG, an ideal advantage conducting NF studies with ADHD subjects [27].

In the current study, we analyzed the differences in connectivity patterns of adults with ADHD between failed and successful NF trials, both in the rest, or preparation phase, preceding the trials and during the trials themselves. NF trials are cognitively active states [1] compared with relatively cognitively-inactive states preceding these trials (resting states). While the difference in brain activation between failure and success in NF has been studied during the course of individual trials in healthy participants [28], to our knowledge there have been no studies evaluating the differences in FC between failed and successful trials, nor in the rests preceding these trials (nor in ADHD). We predicted that, based off of the tendency of subjects with ADHD to have difficulties in switching between cognitively active and restful states, that subjects would show no significant difference in FC between resting periods and active NF, particularly in failed versus successful trials. The NF training analyzed targeted control of prefrontal cortex, therefore we expected enhanced FPCN connectivity during successful NF trials, with less clearly defined patterns during failed trials. During successful rests, we expected connectivity more similar to the DMN, with failed rests displaying FPCN activity due to a failure of task-switching.

However, as we will see in the coming methods section, several problems belonging to the design of the study need to be considered when interpreting the results. Therefore, this paper is divided into two parts: the first discussing the results of the ROI-based FC analysis, and the second offering a critique of, and recommendations for, NF study design.

## Materials and methods

The present data are an excerpt taken from an extensive study [10] comparing functional near- infrared spectroscopy (fNIRS) based neurofeedback with an electroencephalography (EEG) based neurofeedback (for details see [29] and electromyography (EMG) biofeedback training as semi-active control group (for details see [30]). The study (434/2010BO1) was approved by the local Ethics Committee of the Medical Faculty of the University and University Hospital of Tuebingen and conducted according to the ethical guidelines and principles of the international Declaration of Helsinki in its latest version.

### Subjects

Out of the three groups originally comprised in the study design (see above), we only focused on the fNIRS group. 19 adults with ADHD completed 30 sessions of fNIRS–based neurofeedback (age *M* = 30.37 years, *SD* = 9.25; 6 female). Out of the 19 participants, seven were prescribed methylphenidate. All subjects were of the combined ADHD subtype, with the following subscale breakdown on the HASE-Homburger ADHD scale for adults [31]: total symptoms, M = 34.18 (S.D. = 7.43); inattention, M = 17.05 (S.D. = 5.18); hyperactivity, M = 9 (S.D. = 3.69); impulsivity, M = 8.08 (S.D. = 2.37).

### Study procedure

A complete fNIRS–based neurofeedback training was comprised of 33 sessions with one to three sessions per week. Sessions 31–33 were conducted six months after completion of the initial 30 sessions to check for long-term stability of regulation ability and outcome. After 15 sessions, subjects had a three-week intermission and were instructed to practice and implement their acquired feedback strategies in everyday life. In total, mean training duration was 28.61 weeks (*SD* = 9.00; Min/Max = 12.29–49.14). fNIRS measurements (changes in oxygenated hemoglobin concentration elicited by executive functioning tasks), EEG assessments (quantitative EEG, event-related potentials in cognitive tasks) and neuropsychological assessments (symptom ratings, concentration task) were conducted preceding the first session, after 15 sessions, after completion of 30 sessions, and after six months (for details on the complete design, see [10]). Here, we consider only the fNIRS data from the initial 30 training sessions.

### fNIRS neurofeedback setting

Participants sat in front of a monitor in a dark and sound-attenuated room. During the active regulation phases, they received visual feedback reflecting changes in oxygenated hemoglobin (O_2_Hb) in the left and right prefrontal cortex. fNIRS feedback was recorded by means of the ETG-4000, continuous wave system (Hitachi Medical Co., Japan) which was linked to the THERA PRAX® DC-EEG-neurofeedback- and biofeedback system using a DC-EEG- and bio-signal amplifier (neuroConn GmbH, Ilmenau, Germany) and a personal computer. To calculate the input signal for the THERA PRAX,® fNIRS data were fed from the ETG-4000 to the personal computer via TCP/IP protocol for online processing using MATLAB R2011.

**Fig 1:**
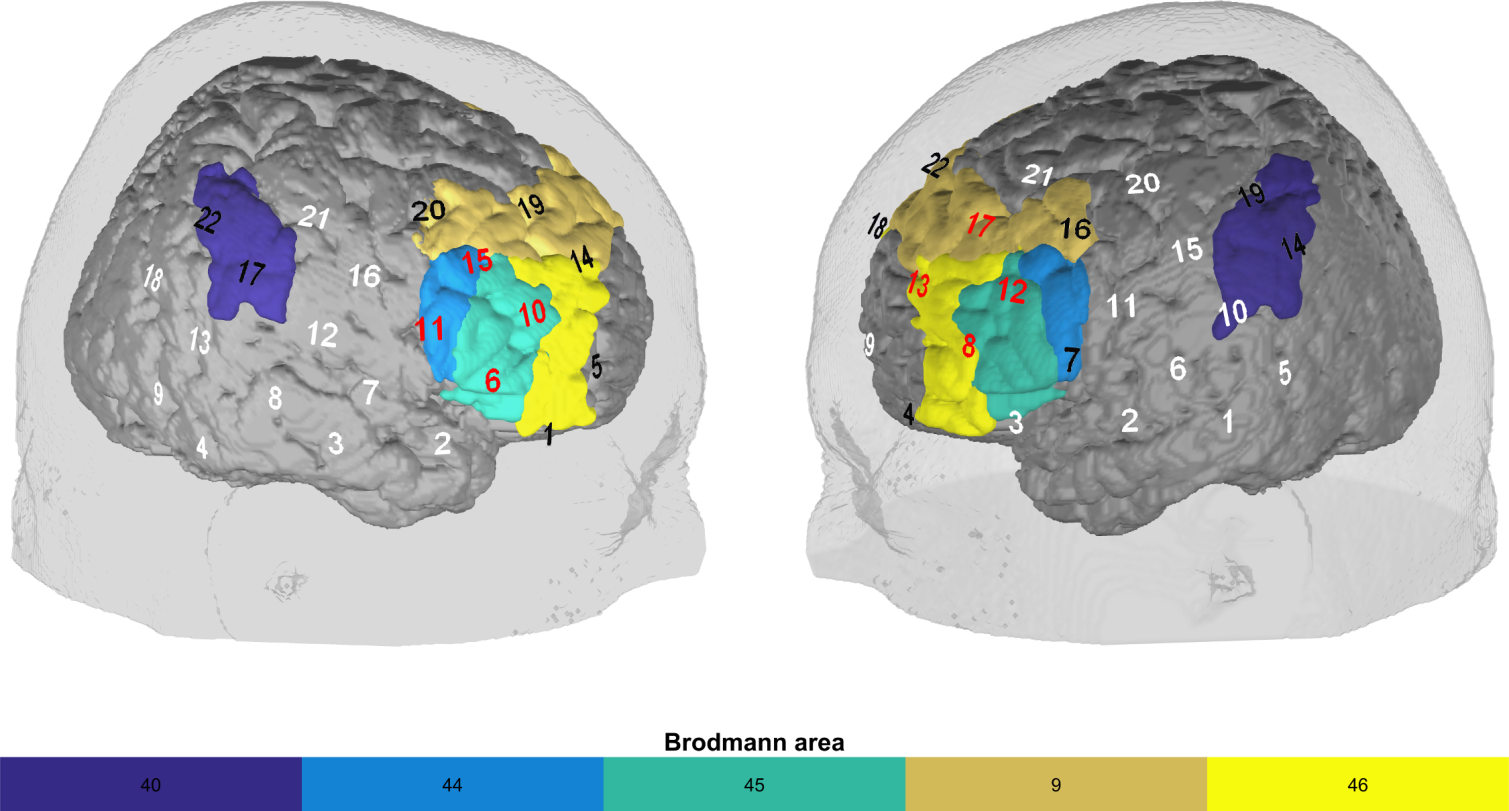
Probeset and regions of interest. The probeset used for the feedback training covered frontal, parietal, and temporal regions. Feedback channels are in red and covered the dlPFC (BA 9, 46) and IFG (BA 44, 45); channels in black are non-feedback channels that were also part of the ROI-based FC analysis. White channels were not included in the analysis. The supramarginal gyrus (BA 40) is also presented, as it is part of the FPCN that we included in the ROI-based FC analysis.

To cover frontal sites on both hemispheres, we used two 3×5 optode probesets (consisting of seven photodetectors and eight light emitters) resulting in 22 channels per probeset, and a total amount of 44 channels (see Fig 1). The interoptode distance was 3 cm. Sampling rate was 10 Hz. Probesets were oriented based on the international 10–20 system of electrode placement [32]. Fpz was marked as mid-point, whileT3 and T4 were used as the positions to place the rearmost channel in the lowest line of the respective probeset. The fNIRS feedback signal was computed online using a common average reference (CAR) to deal with artifacts. The CAR is traditionally used in fNIRS experiments to remove global probeset artifacts such as head motion or arousal-related blood flow [33]. Furthermore, fNIRS and EEG NF experiments commonly employ the CAR to control such artifacts, as it is a computationally efficient method [8,9,34–37]. For each data point during the regulation phase, mean changes in O2Hb of four frontal channels per probeset were calculated. In a next step, the average activity (CAR) of all channels on the respective probeset was subtracted. Finally, the resulting O_2_Hb (feedback) amplitudes for each probeset (four channels on the left and four on the right; see Fig 1) were averaged.

### fNIRS neurofeedback trials

Every session consisted of three blocks of fNIRS-based neurofeedback. Each session lasted approximately one hour including preparation time and was comprised of 32 min of neurofeedback training. One training session included two feedback blocks of 12 regulation trials, each lasting 12 min, separated by an 8 min transfer block comprised of 8 regulation trials. At the beginning of each session, a 10 s baseline measurement was conducted. A feedback block comprised 12 regulation trials lasting 30 s preceded by roughly 25 s resting time and 5 s baseline measurement. The task was either to increase or decrease prefrontal O_2_Hb concentration whereby up-regulation and down-regulation trials were equally likely. At the beginning of every regulation trial, a triangle was presented in the center of the computer screen oriented either upwards or downwards, indicating an activation or deactivation trial (i.e., a required increase or decrease in frontal O_2_Hb concentration), respectively. Visual feedback of relative changes in O_2_Hb was provided by means of an object on the screen which participants could select beforehand (e.g. a moon, a fish). Successful trials (at least 7 s of the last 15 s regulation in the desired direction) were visually reinforced by the symbol of a sun presented on the screen immediately following the trial. During transfer blocks, participants did not receive any continuous visual feedback about prefrontal oxygenation level but received reinforcement for successful trials directly after completion. The transfer condition served as the first step to transferring regulation strategies into daily life, where no direct feedback is given.

### Data analyses

#### Calculation of pre-trial and trial fNIRS neurofeedback data

**Fig 2:**
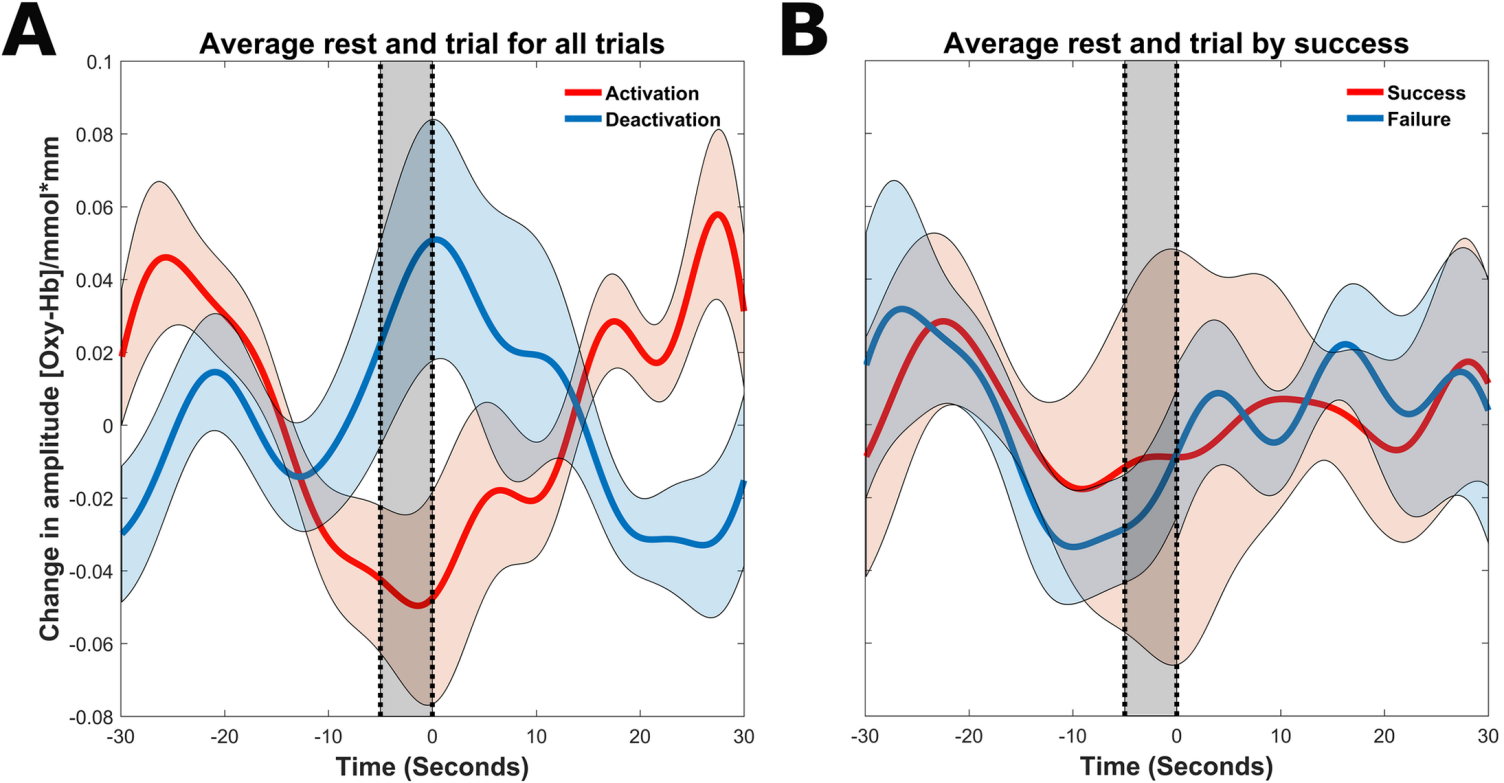
**A**. **Grand average activation and deactivation trials**. Trials are plotted with the standard error of the mean. The black shaded regions represents the baseline time calculated for the feedback trials. **B. Grand average success and failure trials**, averaged over all activation and deactivation trials.

All subsequent data analysis is performed on data from all 30 training sessions. We calculated the average signal within the feedback channels for the 25 s resting period preceding feedback trials and for the 30 s trials for both activation and deactivation trials. The calculation of the average signal was as described in the ‘fNIRS neurofeedback setting’ section with the further averaging of all activation or deactivation trials across all subjects. In the analysis, we used all continuous feedback trials (as opposed to transfer trials) across sessions 1–30. This resulted in a grand average activation and deactivation pre-trial and trial feedback signal (see Fig 2).

#### Calculation of transition probabilities and adjustment of the analysis strategy

With a 50/50 activation/deactivation design, it is important that the probability of switching between an activation and deactivation trial is equal in order to ensure that no pre-baseline biases are introduced into the design. For example, if the likelihood is greater that the kind of feedback trial switches in the next trial (i.e. activation to deactivation or deactivation to activation) then the subject may better prepare during the pre-trial, which might bias what should otherwise be a neutral preparatory phase. Indeed, during the course of analysis, we observed some anomalies in the data that led us believe that activation and deactivation trials had not been properly randomized by the neurofeedback software. Specifically, we observed a clear difference between activation and deactivation trials already in the baseline phase preceding the grand averaged feedback trials (i.e., a baseline bias effect). Namely, before activation trials it appeared that there was a decrease in feedback channel activity just before the beginning of the trial; for deactivation trials it was the opposite, an increase in feedback channel activity just before the trial (see Fig 2). That means, subjects were able to, either intentionally or unintentionally, predict the type of the next trial. With a perfectly randomized design this should not have been possible. Therefore, we decided to investigate the transitional probabilities, or the probability that the next trial type will be the same as the current one (congruent) or the opposite (incongruent).

To this end, we calculated the probability of transitioning from each type of trial to 1) the same type of trial, and 2) the opposite type of trial. To calculate statistical significance, we used a 2x2 repeated measures ANOVA with the within-subjects factors of trial type (activation vs. deactivation) and congruency (incongruent vs. congruent trials). Since this analysis confirmed a systematic bias introduced by improper randomization of trial presentation (see Results section)–and due to the effects such a bias can induce on studies of FC–we decided to analyze combined deactivation and activation trials and their preceding rests and compare success versus failure for differing patterns of FC for the remaining data analysis.

#### Learning rates

In order to calculate the influence of the transition probabilities on the rate of learning for subjects over time we divided the trials into two halves: the first 15 trainings and the last 15 trainings. To calculate the learning rate for each subject, we grouped all activation and deactivation trials together and split them into congruent and incongruent trials. To test statistical significance of differences between halves and congruency, we applied a 2x2 repeated measures ANOVA with the within-subjects factors of time (first vs. second half performance) and congruency (incongruent vs. congruent). We also calculated the learning rates for all trials in the first and second halves and compared them with a paired t-test.

#### Functional connectivity differences between successful and failed feedback trials and the preceding rests

All preprocessing and analysis of FC was computed in MATLAB version 9.0 (The Mathworks Inc., Natick, Mass.) using routines created in our working group. The first step was to segment the trials into successful and unsuccessful trials (30 seconds) and their preceding rests (25 seconds) based on the presentation or lack of a reward. The next step was to recreate the signal that passed in the THERA PRAX® machine. To do this, a moving average was first applied to the raw O_2_Hb data (five second moving window). Next, the common average from each probeset was subtracted from the signal (for more details see the above section fNIRS Neurofeedback trials). Next, the data was bandpass-filtered between .01-.1 Hz to remove potential influence from physiological artifacts. A single trial was created for each subject and each condition by concatenating all continuous feedback trials (across sessions 1–30) of said condition together. In a last preprocessing step before computing FC, a robust outlier detection algorithm was applied to each concatenated trial, removing outliers based on multivariate analysis of covariates and mean. Finally, for each subject a Pearson product-moment correlation was calculated between each pair of channels for the entirety of the trial. This resulted in a single value for each channel pair for each subject for each condition. These values were then normalized with a Fisher’s r-to-Z transformation.

#### Statistical analysis of FC between regions of interest

Statistical analysis was performed in SPSS version 23.0 (IBM Corp, Armonk, NY). We mapped fNIRS channels to corresponding, underlying cortical areas based on a virtual registration method [38–40]. We chose regions of interest (ROIs) based on the composition of the feedback channels and the cognitive control network, which is ultimately responsible for regulating behavior during cognitively demanding tasks such as NF. We had six total ROIs: bilateral dlPFC, bilateral IFG, and bilateral parietal area. In order to test the statistical significance of FC between the regions, we computed an average in the FC between all channel pairs in the defined regions and then averaged these averages, giving one FC value for each regional pairing. We then computed 2x2 repeated measure ANOVAs for each regional pairing with the within-subjects factors of trial type (rest or trial) and success (success or failure). A Bonferroni-Holm correction was applied to resulting p-values to account for multiple comparisons.

## Results

### Grand average pre-trial and trial O_2_Hb activation in neurofeedback channels

For a visual representation, please see Fig 2. When we investigated activation and deactivation trials separately, there was a clear bias introduced in the baseline, wherein for activation trials, the tendency of the feedback signal was to decrease drastically just before the start of the trial. In deactivation trials, the tendency was exactly the opposite, the feedback signal increased (in activation) just before the start of the trial. For both trial types, this affects the ease of achieving the feedback goal in the subsequent trial. Furthermore, it renders a study of FC virtually impossible, as this systematic effect on amplitude bleeds into the FC analysis [41–43]. When we combined all trials and separated based on success or failure, we observed no baseline–dependent effects, and thus a study of FC with combined trial types was possible.

### Transition probabilities

Based on the above-reported findings of unexpected baseline differences in the fNIRS/feedback signal, we analyzed the transition probabilities between trials in detail. A 2x2 repeated measures ANOVA revealed that there was indeed an effect of transition type (*F*(1,18) = 126.33, p < .001, η^2^ = 0.875). The likelihood of switching to the opposite trial type (incongruent trials) was significantly higher than staying with the same trial type (congruent trials) (M_switch_ = .395, SD = .01; M_non-switch_ = .324, SD = .01). There were no other significant main effects or interaction effects.

### Learning rates

**Fig 3:**
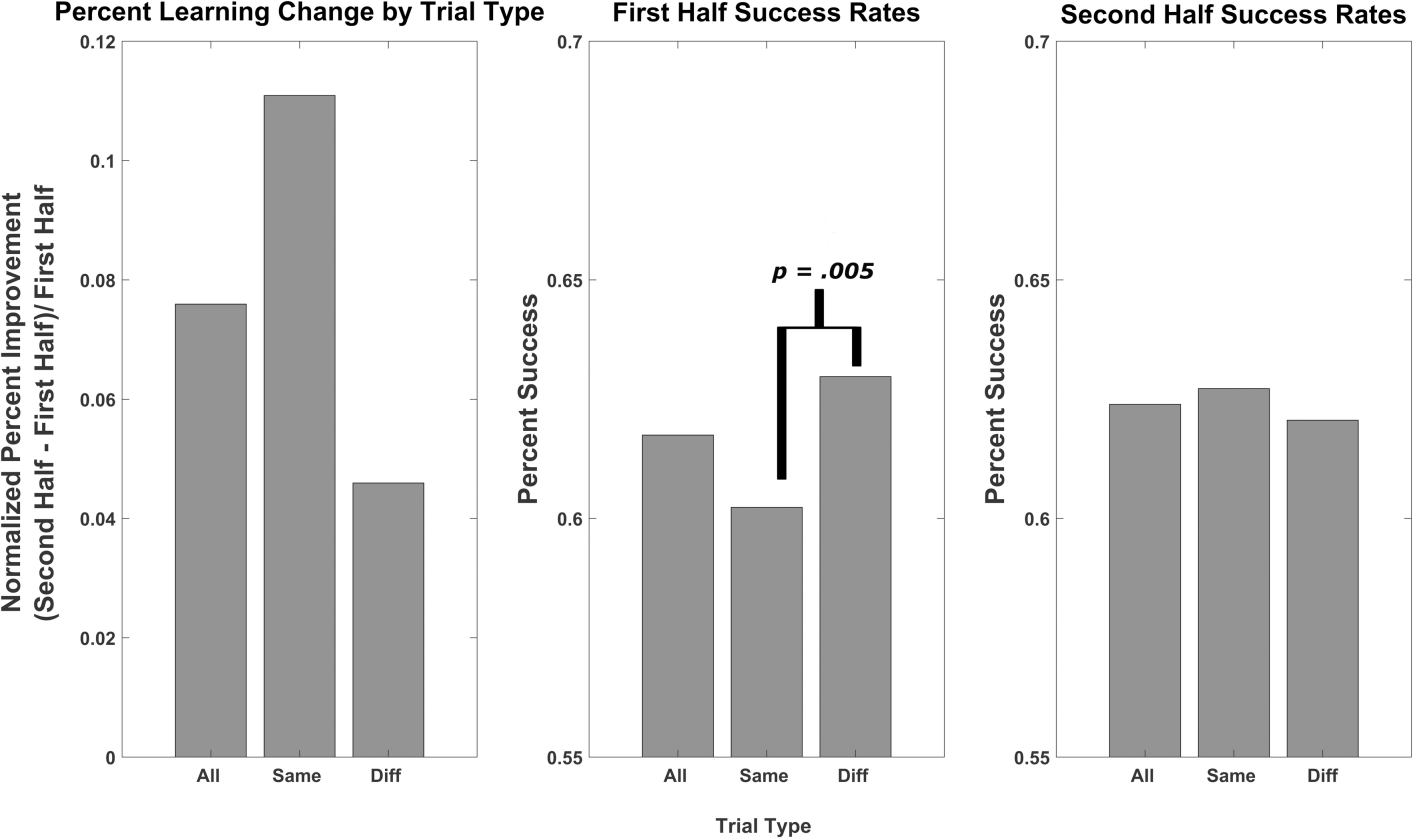
Learning rate between first and second half performance. The learning rates for all, congruent and incongruent trials are depicted in the left graph. The middle and right graphs depict the first and second half performances, respectively. All activation and deactivation trials were grouped together.

Learning rates showed no main effect of time or trial congruency (all *F*(1,18) < 1.22). There was a significant interaction effect of time*congruency (*F*(1,18) = 9.33, p = .007, η^2^ = 0.82). Incongruent trials, which were significantly more likely to occur than congruent trials, were also statistically more successful in the first half of sessions than congruent trials (M_incongruent_ = .630, SD = .132; M_congruent_ = .602, SD = .136, *t*(18) = 3.222, p = .005). This effect disappeared in the second half of sessions (M_incongruent_ = .627, SD = .121; M_congruent_ = .620, SD = .097, *t*(18) = .592, p = .561). There were no differences in all trials from the first to the second halves (M_first_ = .618, SD = .134; M_second_ = .624, SD = .103, *t*(18) = .154., p = .879). These results are depicted in Fig 3.

### ROI-based FC

**Fig 4:**
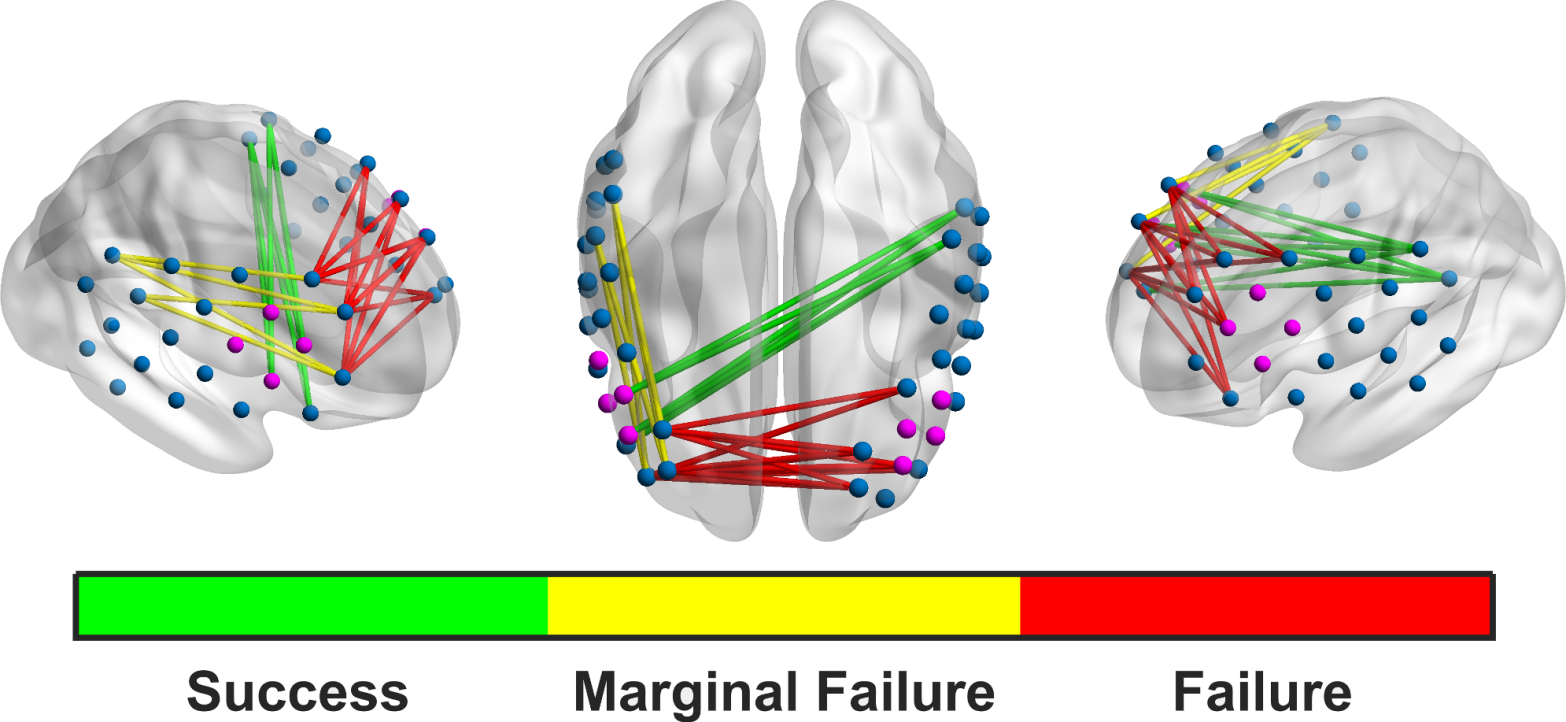
Significant ROI-based connectivity plots. The green Success FC occurs during successful rests and trials and is between the right IFG (making up a considerable part of the feedback channels) and left parietal lobe. The red Failure FC is between the left and right dlPFC, and is stronger on failed trials and rests. Connectivity between the right dlPFC and right parietal lobe is marginally stronger during failed rests and trials than successful ones.

After correcting for multiple comparisons, there was a significant main effect of success for connectivity between the left and the right dlPFC (*F*(1,18) = 27.05, p < .001, η^2^ = 0.600). FC was higher in the bilateral dlPFC during failed as compared to successful trials and rests (M_success_ = .208, SD = .122; M_fail_ = .235, SD = .123). There was also a significant main effect of success for FC between the right IFG and the left parietal ROI (*F*(1,18) = 15.08, p = .014, η^2^ = 0.456). FC was higher between the regions during successful trials and rests (M_success_ = .128, SD = .100; M_fail_ = .116, SD = .096). There was also a marginally significant main effect of success for FC between the right dlPFC and the right parietal ROI (*F*(1,18) = 9.63, p = .091, η^2^ = 0.342). FC between these two regions was higher during failed trials and rests than successful ones (M_success_ = .185, SD = .129; M_fail_ = .207, SD = .128). There were no significant main effects for trial type or for interactions between trial type and success (see Fig 4).

## Discussion

This paper–and particularly the subsequent discussion of our findings–is split into two parts: the first part addresses FC within a NF experiment for adults with ADHD, while the second part addresses the complex issue of designing a NF experiment.

In the FC analysis, we observed significant patterns of increased bilateral dlPFC connectivity and marginally significant patterns of increased FC between the right dlPFC and the right parietal ROIs during failed rests and trials. In contrast, we observed increased right IFG to left parietal connectivity in successful rests and trials. The “success network” involved significantly stronger connectivity between the right IFG and the left parietal regions. Both regions are involved in the FCPN [44], so a concurrent activation makes sense during a cognitively active task. In the “failure network(s)”, the right dlPFC is centrally involved in both significantly stronger bilateral dlPFC FC and also marginally stronger FC with the right parietal ROI during failed trials and rests. Normally, the bilateral dlPFC is integrally involved in the FPCN [45–47]. Spreng et al. (2013) suggest that the dlPFC may actually be a common link between the Dorsal Attention Network and the FPCN. Furthermore, they found no FC between the dlPFC and the DMN, indicating once again its pivotal role in cognitively complex tasks. Sridharan et al. (2008) found that the right frontal insular cortex, an area that coactivates with the right dlPFC, possibly functions as a switching region, easing transition between the DMN and the FPCN. Of particular interest is that the right feedback channels are completely comprised of the right IFG. Because the right dlPFC was not involved in the feedback calculation, it may be that its activation was not encouraged, potentially hindering successful switching between rest and cognitively active trials. Furthermore, any strong activation (or deactivation in deactivation trials) in the right dlPFC (as well as other elements of the FPCN not involved in the feedback channels) would actually lower (or raise in deactivation trials) the average feedback activation due to the CAR being subtracted out. Therefore, when subtracting the CAR, the right IFG-left parietal iteration of the FPCN was the least affected. This shows us two things: first, using the CAR to isolate (training of) a particular region may not work as we intended. It appears that network activation is still influencing successful trials. Furthermore, the CAR may actually be punishing activation of other parts of the FPCN. Because of this, the right IFG-left parietal connectivity observed during successful trials emerged as the best option for activating the FPCN to achieve a successful trial given the design of the current experiment. Interestingly, in our FC analysis, there were no significant differences between rest and trials, rather only between success and failure. The successful NF trial, then, may be dependent on network interplay that begins first during the resting phase and continues into the actual feedback trial, particularly with the problems that we will soon discuss. This makes sense when we consider the resting phase to be more akin to a preparation phase for the upcoming trial. The lack of difference between rest and trial may also reflect the difficulty that subjects with ADHD have in switching between cognitively active and inactive states [18]. However, without a proper healthy control group, we cannot confirm this.

When we consider NF experimental design, we need to consider what we actually train when using a CAR in the algorithm: is the focus more on the desired training parameter or on the activity being subtracted out by the CAR? The debate surrounding the CAR stems initially from EEG research. In EEG, the CAR produces problems similar to what we observe in this NIRS experiment, but for different reasons. Nunez and Srinivasan [48] stress that the locally recorded EEG signal is always dependent on the distal reference. With a CAR, distal effects due to volume conduction will necessarily taint the true nature of the local signal, although the global artifacts will be reduced, resulting in a higher signal to noise ratio [49]. In the realm of NIRS, many studies have used or use a CAR to reduce system-wide influences, such as respiration, heartbeat and motion artifacts, on the brain signal [8,9,34,50]. Three of these studies are NIRS-based NF studies dealing with ADHD or impulsivity. These are all studies in which the CAR punished potentially helpful network activity. Nevertheless, Hudak et al. [34] and Marx et al. [9] realized beneficial results for highly impulsive and ADHD populations, respectively. It is therefore unlikely that the CAR diminished all network activity, but instead forced the network to operate differently. Still, optimization of the feedback algorithm might then allow for even better results.

The systematic bias in the pre-trial baseline of the feedback channels for both activation and deactivation trials is another important NF design consideration. An uneven distribution of transition probabilities between trial types likely caused this bias. It was significantly more likely for incongruent trials to occur than congruent ones. Therefore, the subjects were able to prepare themselves for the upcoming trial; whether this was conscious or unconscious cannot be determined from this study. Furthermore, this bias led to significant performance differential between congruent and incongruent trials in the first half. Subjects were more likely to succeed on the more probable incongruent trials than on their congruent counterparts. This differential disappeared in the second half, perhaps due to subjects having more experience with the less common congruent condition. As can be seen in Fig 3, the less common, and therefore more difficult, congruent condition converges on the more common incongruent in the second half. However, from our dataset, it is not possible to conclude whether the ‘failure network’ that we identified in the FC analysis comes is linked to failed performance, or to a mismatch between what was expected and what actually happened because of the bias. Because of this bias, and its potential influence on FC, we decided to combine the activation and deactivation trials into one analysis. Interestingly, as a result of combining the trial types before the FC analysis, we show the role of the FPCN in both activation and deactivation trials. Just as the network must activate in unison to achieve success on activation trials, it must deactivate in unison to achieve deactivation success. The FC, then, should not differ between the two types.

When we combined activation and deactivation trials, we observed no pre-baseline differences in successful versus failed feedback trials, and so a comparison of FC patterns in success and failure was possible. In the present study, this bias likely affected FC nevertheless, as the preparation in the resting phase may have blended into the active trial state. While this pre-trial baseline bias may help subjects to achieve better rates of success on the actual feedback trials, it is troublesome for a few reasons. Firstly, when subjects are learning to regulate particular brain parameters, they are usually doing so implicitly through trial and error [51]. An experimental design that encourages deactivation before activation trials and activation before deactivation trials may only be training the timing of the natural neuronal signals and not actively encouraging increasing or decreasing of the intended parameter [52]. Furthermore, FC analysis is particularly influenced by amplitude changes in the signal [41,43]. When participants rapidly change the signal in anticipation of certain trial types, this can have a strong impact on connectivity, particularly when the window is short (i.e. 30 seconds or less). FC is based off of signal deviance from individual means; therefore, in a short window of calculation, these amplitude spikes will produce greater inflations in FC [42].

The reasons for the baseline bias are clear. There was a significantly greater probability of switching to a different trial type, and the baseline calculation for the coming trial was calculated as the average of the last five seconds of feedback channel activity. In isolation, either of these problems would not lead to drastic effects on the feedback trials themselves, but in combination, it produced the observed pre-baseline bias. A clear practical recommendation when moving forward is to always pseudo-randomize the trial presentation so that there is an equal chance of all trial types being next. This is a simple, but often overlooked, factor in study design. For example, the commercial NF machine used in our study is very regularly used in both scientific research and clinical practice. Very few studies analyze or report on this factor, though potentially all corresponding studies and treatments could benefit from controlling the randomization of trial type.

Otherwise, one could employ a non-local baseline calculation, or simply a much longer one that considers the entire trial. A universal baseline in the beginning of the experiment has the advantage of not being beholden to artifacts induced by local movements or spontaneous signal fluctuations, but it is more susceptible to delivering poor results in the experiment over time, as the signal is prone to drifts over time and also to displacement due to larger movements. One option that may be preferable to the local baseline calculation is to use a reference condition or a block built into the NF session. All subsequent NF trials are then compared to this reference trial instead of a baseline, thereby avoiding the pitfalls associated with local baseline bias [53,54].

One limitation of the current study was that 30 percent of the participants were taking Methylphenidate. Asking participants to cease their intake of Methylphenidate is not advisable for a study spanning such a long time-period. The effects of Methylphenidate on the BOLD response are not entirely conclusive, as depending on the brain area involved, it can both hinder and increase the response [55,56]. As this was a within-subjects analysis, the effects of Methylphenidate should have been constant throughout the entire 30 trainings.

## Conclusions

In conclusion, the current study reflects several small but significant factors that have strong influences on NF design. Improper randomization or intentional unbalancing of NF trial type may cause unintended bias in the pre-feedback resting phase, particularly if paired with a local baseline. Even more potentially disruptive, the CAR needs to be carefully considered before introducing it into a feedback design that is dependent on network activity. One way forward would be to consider subtracting activity from a region of channels not connected to expected cognitive control networks. Of course, FC based-NF designs would also be a great way to target ADHD dysfunction.
